# Down regulation of PSA by C/EBPα is associated with loss of AR expression and inhibition of PSA promoter activity in the LNCaP cell Line

**DOI:** 10.1186/1471-2407-6-158

**Published:** 2006-06-14

**Authors:** Hong Yin, Hanna S Radomska, Daniel G Tenen, Jonathan Glass

**Affiliations:** 1 Feist-Weiller Cancer Center and Department of Medicine, LSU Health Sciences Center, Shreveport, LA, USA; 2 Harvard Institute of Medicine, Beth Israel Hospital, Boston, MA, USA

## Abstract

**Background:**

C/EBPα is a transcription factor essential for terminal differentiation of several cell types. It has not known if C/EBPα protein is expressed and functions in the prostate gland.

**Methods:**

The presence of C/EBPα in normal and cancerous prostate epithelium was examined by immunochemistry. Over expression of C/EBPα in LNCaP cells was conducted with retrovirus-mediated transduction. PSA expression was examined by RT-PCR and western blot and PSA promoter activity by luciferase reporter assay.

**Results:**

In normal prostate C/EBPα was expressed in the basal layer of the epithelium. In prostate cancer C/EBPα was detected at low levels throughout the cancers and in advanced prostate cancer C/EBPα expression was associated with decreased expression of AR and PSA. Overexpression of C/EBPα inhibited epigenetically PSA expression and was accompanied by the loss of expression of AR. Transient increase of C/EBPα inhibited the PSA promoter/enhancer activity independently of expression of AR.

**Conclusion:**

In LNCaP cells C/EBPα over expression inhibits expression of PSA by AR -dependent and independent mechanisms and by extinguishing AR expression provides a model for hormonal independent cell growth.

## Background

The CCAAT/enhancer binding proteins (C/EBPs) are a family of transcription factors that regulate cellular differentiation in a variety of tissues [1]. The C/EBP transcription factors consist of an activation domain, a DNA-binding basic region, and a leucine-rich dimerization domain. The structure of C/EBPα and its isoforms has been well described. The predominant isoform is a 42 kDa protein with three transactivation domains in the N-terminal portion of the protein. A second isoform is a 30-kDa protein formed from a downstream initiation site that alters the transactivation domain. Both isoforms contain the same dimerization and DNA-binding domains and are influential in controlling terminal differentiation and growth of a variety of cells [2,3]. C/EBPα is expressed in numerous tissues with the highest expression in the placenta, liver, lung, skeletal muscle, pancreas, small intestine, colon and peripheral blood leukocytes. The C/EBPs in general are important regulators of cellular differentiation and determinants of terminal cell function. C/EBPα in particular is required for myeloid differentiation; mice deficient in C/EBPα have an early block in granulocyte maturation with a lack of expression of the G-CSF receptor[4,5]. The role of C/EBPα in hematopoietic differentiation is complex as at the same time that C/EBPα induces granulocyte differentiation while erythroid differentiation is inhibited [6]. C/EBPα plays a role in hepatocytes and adipocytes mediating cell cycle arrest and transcription of hepatic-specific and adipose-specific genes respectively. C/EBPα interacts with regulators of cell cycle expression such as cdk2 and cdk4, directly interacts with E2F, and represses c-Myc expression with a resultant inhibition of adipocytes, hepatocyte, and granulocyte proliferation and induction of terminal differentiation [7-9]. In view of the expression of C/EBPα in terminally differentiated tissues and its role in regulating cell growth it is attractive to examine whether abnormalities of expression of C/EBPα have a role in carcinogenesis. Recently, C/EBPα has been shown to be important in a subset of patients with acute myelogenous leukemia. In patients with a t(8;21) translocation the resulting AML1-ETO fusion protein blocks C/EBPα expression and restoration of C/EBPα expression restores granulocyte differentiation [10]. In other patients with morphologically similar leukemias, but without the t(8;21) translocation, heterozygous mutations in C/EBPα result in the production of an amino terminus truncated protein that acts in a dominant negative manner to block C/EBPα DNA binding [2]. The resulting lack of transactivation of granulocyte target genes gives rise to the characteristic loss of differentiation seen in acute myelogenous leukemia.

The presence of C/EBPα in normal prostate tissue [11] and the role of C/EBPα in the regulation of differentiation and growth in other tissues raises the issues as to whether C/EBPα is important in normal prostatic differentiation and if aberrations of expression of C/EBPα are seen in prostate cancer. Significant levels of C/EBPα mRNA can be detected in the prostate [11,12]. While only one silent mutation of C/EBPα has been found in 33 prostate cancers [13] increased expression of C/EBPα RNA in cancerous prostate epithelium versus normal epithelium and stroma has been detected [14]. To understand the function of C/EBPα in the prostate we examined first the distribution of C/EBPα in normal and cancerous prostatic epithelium. We then examined the effects of C/EBPα on the expression of the prostate specific antigen (PSA). PSA is a serine protease and a member of the kallkrein family. In normal prostate, PSA is produced in secretory epithelial cells to liquefy semen [15] and PSA is considered a marker of prostate epithelial differentiation [16,17]. In prostate cancer, PSA is an important biomarker for the diagnosis, treatment evaluation, and prognosis [18,19]. An elevated serum PSA often indicates the presence and/or recurrence of prostate cancer [20] and high levels of serum PSA is typical of patients with hormone refractory prostate cancer. In addition to semen liquefaction, PSA is thought to regulate prostate proliferation through the cleavage of insulin growth factor binding protein-3 (IGFBP-3) to activate IGF signaling [21-23]. Recent studies showed that PSA may contribute to bone metastasis by enhancing adhesion of prostate cancer cells to bone marrow endothelium and activating latent TGFβ 2 [24]. The expression of PSA is mainly regulated by the androgen receptor (AR) [15,25,26]. Inhibition of AR signaling blocks the expression of PSA. The tumor suppressor genes PPAR gamma and p53 inhibited PSA expression through AR signaling [27,28]. PSA is also regulated by androgen-independent mechanisms [29]. In this study, we demonstrated the inhibition of PSA expression by C/EBPα via epigenetic loss of AR expression and direct interaction between C/EBPα and the PSA promoter/enhancer.

## Methods

### Cell culture and cell transduction by retrovirus expressing C/EBPα

The human prostate cancer cell line, LNCaP (ATCC, Rockville, MD), was maintained in RPMI 1640 medium (Mediatech Cellgro, Herndon, VA) supplemented with 10 % fetal bovine serum (FBS). LNCaP cells with stable expression of C/EBPα were established with a pantropic retroviral expression system (BD Biosciences Clontech, Palo Alto, CA). Briefly, the full length rat C/EBPα cDNA which shares 94 % homologuos to human C/EBPα was inserted into the retrovirus vector pLNCX and was co-transfected with Lipofectamine Plus (Invitrogen Life Technologies, Inc., Carlsbad, CA) into GP2-293 packaging cells with pVSV-G, expressing an envelope glycoprotein of the vesticular stomatitis virus. After 48 hours of transfection, medium was collected, filtered, and prostate cancer cell lines were transduced with a mixture of virus-containing medium and fresh medium at ratio of 1:2. Polybrene (Sigma-Aldrich, St. Louis, MO) was added to the medium at 8μg/ml for first 24 hours. Stable expressing clones were selected with geneticin at 400 μg/ml (Mediatech Cellgro, Herndon, VA) for 2–3 weeks.

### Immunohistochemistry and immunofluorescence

Immunohistochemical staining of the tissue slides was performed by deparaffinization, treatment with 4% H_2_O_2 _to remove endogenous peroxidase activity, and antigen retrieval with 10 mM sodium citrate solution. After blocking the samples with 15% fetal bovine serum in phosphate buffer saline (PBS) for 30 min the sections were incubated at room temperature with antibodies against C/EBPα antibody (sc-61), androgen receptor (sc-7305), and PSA (sc-7316) (Santa Cruz Biotechnology, Inc., Santa Cruz, CA) or against p63 (Lab Vision Co., Fremont, CA). The immunohistochemical staining was performed with multi-link reagent and AEC substrate (BioGenex, San Ramon, CA). For immunohistochemical staining of human prostate tissue, archived samples were obtained from the Department of Pathology of LSUHSC under a LSUHSC IRB approved protocol (Protocol No:E06-110).

### Luciferase reporter assay of PSA promoter/enhancer activity

Plasmids containing the PSA promoter and enhancer driving the luciferase reporter gene were the kind gift of Dr. Stephen P. Balk. One plasmid contained the proximal promoter, the distal enhancer from -6480 to +12 nucleotides, and had multiple androgen receptor elements (ARE). The other plasmid contained the proximal promoter from -632 to +12 nucleotides and had two ARE sites[25,26]. Prostate cancer cell lines were grown in 24 well plates to about 70–80% confluence and were co-transfected either with 375 ng PSA promoter/enhance or proximal promoter-driven luciferase reporter plasmid and 0–100 ng pcDNA3-rat C/EBPα expression vector or mutated C/EBPα [2] using Lipofectamine Plus according to manufacturer's instruction. The cells were placed in medium with charcoal stripped FCS, 50 nM dihydrotestosterone added, and after 36 hours, the transfected cells were lysed with Passive Lysis Solution (Promega, Madison, WI). The luciferase activity was measured with the Dual-Luciferase^® ^reporter assay system (Promega, Madison, WI) on a Monolight 2010 Luminometer (BD PharMingen, San Diego, CA). Renilla luciferase activity was used to normalize the transfection efficiency. The statistical significance was calculated with the unpaired Student *T*-test two-tailed analysis.

### Cell sorting and RT-PCR

The C/EBPα-EGFP plasmid was constructed with a full length rat C/EBPα cDNA in the pcDNA3 vector. Briefly, pcDNA3-C/EBPα was digested with BamH1 and Sac1 and the insert containing C/EBPα was subcloned into pEGFP-N2 vector at Bgl2 and Sac1 sites. LNCaP cells were transiently transfected with C/EBPα-pEGFP by electroporation. After 48 hours, the cells were collected by trypsinization and sorted with the BD FACSVantage™ SE System at λ_ex_488 and λ_em_522. The RNA was isolated from the sorted population of fluorescence-positive cells. RT-PCR was used to detect PSA mRNA expression. One μg of RNA was reversibly transcribed with Superscript™ II, RNase H- Reverse Transcriptase (Invitrogen, CA) and PCR was conducted with HotStarTaq DNA Polymerase (Qiagen, Valencia, CA) using RT product diluted at a ratio of 1:10. The primer pairs used for PSA and glyceraldehyde-3-phosphate dehydrogenase (GAPDH) were, 5'-GGTGATGACTCCAGCCACGA-3'(PSA forward), 5'-GCGCACACACGTCATTGGAA-3' (PSA reverse), 5'-CTACTGGCGCT GCCAAGGCT-3' (GAPDH forward), and 5'-GCCATGAGGTCCACCACCCTGT-3' (GAPDH reverse). The conditions for the PCR were set at 30 s at 95°, 50 s at 57°C, and 1 min. at 72°C for 32 cycles following 15 min. at 95°C at the beginning of the PCR procedure to activate the hot-start taq DNA polymerase.

### Western blot analysis

Whole cell extracts from prostate cancer cell lines were obtained with RIPA buffer (PBS, 1% Nonidet P-40 (NP-40), 0.25% sodium deoxycholate, 0.1% sodium dodecyl sulfate (SDS)) containing 1X protease inhibitor cocktail (Roche Applied Science, Indianapolis, IN). Protein concentration was determined by BCA Protein Assay Kit (Pierce Biotechnology, Rockford, IL). Cell proteins were separated by electrophoresis on 12% SDS-PAGE, transferred to Hybond ™ ECL nitrocellulose membrane (Amersham Biosciences, Piscataway, NJ), and blocked with 5% non-fat milk in 1X TBST (10 mM Tris-HCL, pH 8.0; 150 mM NaCl; 0.05% Tween-20). The blots were then incubated at room temperature with rabbit anti C/EBPα antibody for 2 hours, washed, and incubated with peroxidase-conjugated secondary antibody. The signal was detected with SuperSignal West Pico Substrate (Pierce Biotechnology, Rockford, IL). Antibodies against C/EBPα (sc-61), androgen receptor (sc-816 and sc-7305), PSA (sc-7638) were purchased from Santa Cruz Biotechnology, Inc.(Santa Cruz, CA). The antibody against the β-tubulin (MS-719) was from Lab Vision Co. (Fremont, CA).

### Cell growth analysis

Growth curves were generated by plating the retrovirus infected cells suspended in RPMI 1640 containing 10% FBS and 400 μg/ml of geneticin in 12-well plates at initial cell densities of about 40,000 cells/well. Every two days cells were collected from triplicate wells by trypsinization. The cells were then resuspended in Isoton^® ^II (Beckman Coulter Company, Hialeah, FL) and counted on a Coulter Z1 (Beckman Coulter Company, Hialeah, FL).

## Results

### Immunohistochemical identification of C/EBPα in normal and cancerous prostate cancer

**Figure 1:**
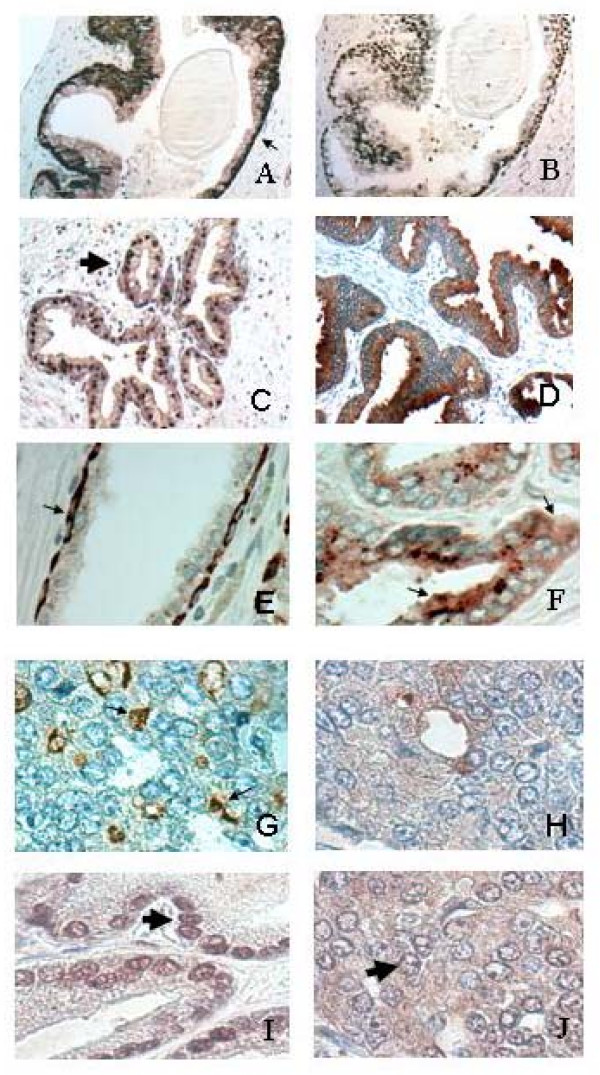
Localization of C/EBPα, p63, AR and PSA in normal and cancerous prostate epithelium. Tissue sections of human prostate glands were stained with antibodies to C/EBPα, p63, AR and PSA as detailed in the Methods. Shown are representative fields of normal and hyperplastic epithelium (panels A-E) and regions with cancer (panels F-J). Panels A, E, F, and G were stained for C/EBPα, Panels D and H were stained for PSA, Panel B was stained for p63, and Panel C, I, and J were stained for AR. Panels A-D are shown at low magnification (10x objective) and Panels E-J at high magnification (40x objective). The thin arrows indicate representative areas staining positively for C/EBPα in normal and cancerous regions. In the normal or hyperplastic epithelium C/EBPα is detected in the basal layer. In Panel D strong staining of PSA is seen at the abluminal surface of the normal prostate epithelium while in Panel H weakly stained PSA is localized diffusedly throughout the cancerous epithelium. In panel I the thick arrow indicates cells staining positively for AR. In Panel J weak staining of AR is seen in some nuclei.

In studies of altered gene expression in cancerous versus non-cancerous prostrate epithelium, increased expression of C/EBPα has been identified at the mRNA level in about 30 % of prostate samples [14]. To determine if altered expression of C/EBPα also occurred at the protein level the presence of C/EBPα was examined by immunohistochemistry in tissue slides from 21 different cases of prostate adenocarcinoma. In normal or hyperplastic glands C/EBPα was detected predominately in the basal cell layer (Figure 1A and 1E). The expression of C/EBPα in the normal glands showed a similar distribution to p63, which is a marker of basal cells of prostate gland and potential marker of prostate stem cells (Figure 1B). As markers of prostate differentiation the expression of AR and PSA was predominately localized to secretory epithelial cells at the abluminal surface (Figure 1C and 1D). In nineteen of the 21 prostate cancer cases C/EBPα was detected albeit with variable intensity from case to case (Figure 1F and 1G) and often with an intensity of staining less than seen in the basal layer of normal or hyperplastic glands. In addition, cells staining strongly for C/EBPα were often seen in the top layer of the cancerous epithelium forming pseudo lumen. In the 19 cases with positive staining for C/EBPα, the staining was seen regardless of Gleason score. For example, in a case with Gleason score 4+5 significant amounts of C/EBPα were detected in at least 10 to 15 % of the cancer cells (Figure 1G). Advanced cancerous regions often displayed apparently lower amounts of AR and PSA expression (Figure 1J and 1H) compared with AR and PSA expression in hyperplastic glands (Figure 1I and 1D).

### C/EBPα expression inhibits expression of AR and PSA

**Figure 2:**
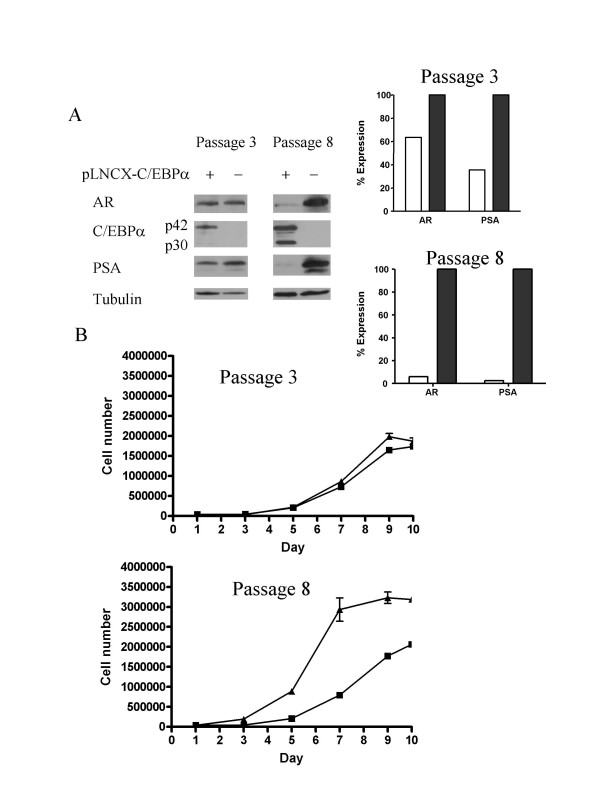
Down-regulation of the expression of AR and PSA after expression of C/EBPα in LNCaP cells. A. As described in the Methods whole cell extracts were obtained from LNCaP clone 2 cells at passage 3 and passage 8 and from corresponding control cells, subjected to SDS-PAGE and western blot analysis for AR, PSA, C/EBPα, and β-tubulin. The two right panels show the relative expression of AR and PSA at passage 3 and passage 8 after densitometry analysis of the western blot was standardized to β-tubulin expression. The open bars represent the Clone 2 cells expressing C/EBPα and the dark bars, the corresponding control LNCaP cells transduced with vector alone. B. As described in Methods growth curves were obtained for Clone 2 LNCaP cells transduced by retrovirus carrying C/EBPα (■) and control virus (▲) at passage 3 and passage 8.

The immunohistochemistry findings in Figure 1 indicated that C/EBPα was present in the in the basal layer of normal prostate epithelium while AR and PSA were localized to the differentiated secretory epithelial layers. What then is the role of expression of C/EBPα in the basal cells of normal prostate epithelium? In contrast to the known role of C/EBPα in other tissues is the expression of C/EBPα inhibiting differentiation of basal cells to secretory epithelium? And in the cancerous tissue is the expression of C/EBPα affecting differentiation and proliferation? To begin addressing these questions we tested the effect of C/EBPα expression on AR and PSA expression in LNCaP cells, a prostate cancer cell line that expresses both AR and PSA. The constitutional expression of C/EBPα in the LNCaP cells is extremely low. Hence, we introduced the C/EBPα gene into the cells by a retroviral construct carrying the rat C/EBPα cDNA. After selection by geneticin, two pooled clones with constitutional expression of C/EBPα were established from the LNCaP parental cells. The nuclear localization of the expressed C/EBPα was demonstrated by the western blot analysis of nuclear extracts from the two clones (data not shown). In clone 1 C/EBPα extinguished expression of AR and PSA immediately. Interestingly, in clone 2 with repeated passage the cells displayed increasing expression of C/EBPα and decreasing expression of AR and PSA (Figure 2A). By the third passage AR and PSA expression had decreased to 37% and 64% respectively of control cells transduced with virus vector alone and by the eighth passage expression of AR and PSA was almost non-detectable. In addition with sequential passages of clone 2 cells C/EBPα expression increased 1.6 fold between passage 3 and 8. Unexplained is the increased expression of the p30 isoform of C/EBPα in clone 2 at passage 8 while in clone 1 cells the p30 isoform was not expressed (data not shown). The long term expression of C/EBPα decreased not only the expression of AR and PSA but also significantly stimulated cell proliferation. The C/EBPα-expressing cells at passage 8 showed more rapid growth than the control cells (Figure 2B). To Confirm the cell proliferation by long term expression of C/EBPα in prostate cancer cells, we introduced the virus carrying C/EBPα cDNA into PC3 and DU145 cells and the increased cell proliferation was also seen in the two transduced cell clones (data not shown)

**Figure 3:**
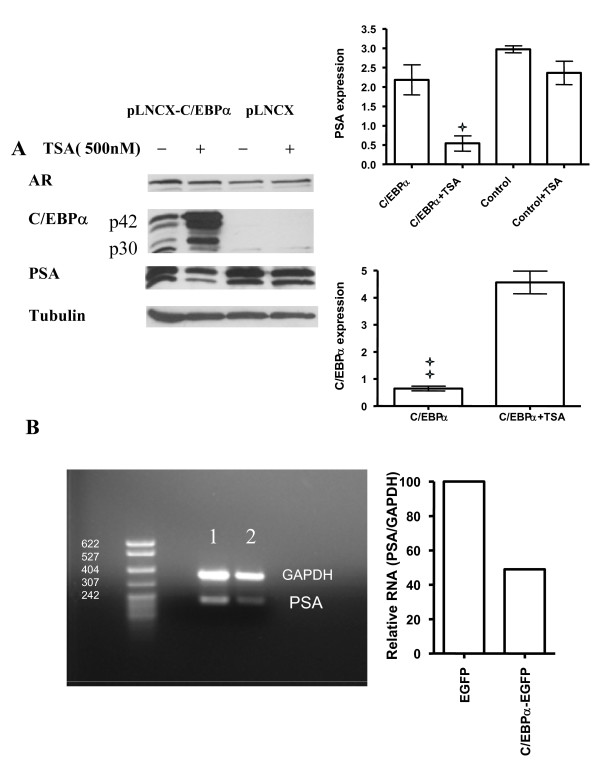
Expression of C/EBPα inhibits PSA expression through inhibition of the PSA promoter. **A**. Increasing expression of C/EBPα reduced protein levels of PSA in transduced LNCaP cells. The Clone 2 cells at passage 3 and the corresponding control cells were treated with 500 nM trichostatin A (TSA) for 48 hours. Whole cell protein extracts were again subjected to SDS-PAGE and western blot analysis performed. The expression of PSA and C/EBPα were expressed relative to β-tubulin by densitometry of the western blots. The results in the right panels are the means ± standard deviation of three independent experiments. For PSA expression a single star indicates a significant difference with a P < 0.05 (unpaired t-test) comparing C/EBPα to C/EBPα plus TSA. For C/EBPα expression double stars indicate a significant difference with a P < 0.01 (unpaired t-test). **B**. LNCaP cells were transiently transfected either with pEGFP-N2, the transfection control, or C/EBPα-pEGFP. After 48 hours EGFP positive cells were collected by cell sorting and RNA extracted from the EGFP positive cells. The PSA mRNA was amplified with RT-PCR as detailed in the Methods. In the left panel is shown the electrophoresis pattern of the PCR products from the cells transfected with pGFP-N2 (lane 1) and C/EBPα-pEGFP (lane 2). A densitometric scan of the electrophoretic pattern corrected for levels of GAPDH shows the relative amounts of PSA mRNA in the control versus C/EBPα transfected cells (right panel) with the columns in the right panel representing the means of two individual PCR reactions.

The extinguished expression of AR and PSA could either be separate phenomena or, as the PSA promoter is known to have multiple androgen receptor response elements (ARE), if C/EBPα decreased AR expression then PSA expression would decrease also. To test the two possibilities we first examined the effect of further increases of C/EBPα on AR and PSA expression. To increase C/EBPα expression we exposed the clone 2 cells to trichostatin (TSA), an inhibitor of histone deacetylase, to activate retrovirus-mediated gene expression. The addition of TSA to the clone 2 cells for 48 hours resulted in a 7 fold increase of C/EBPα protein (Figure 3A). Concomitant with the increase of C/EBPα PSA expression decreased by 75 % but AR levels remained unchanged. We then examined the effect of transient transfection of C/EBPα on endogenous PSA expression in LNCaP cells transfected with the C/EBPα-pEGFP construct. Using a construct that expressed EGFP allowed the transfected cells to be sorted by flow cytometry. In the transfected cells the levels of PSA mRNA in the C/EBPα-pEGFP expressing cells (Figure 3B, lane 2) decreased by approximately 2.5-fold compared to the cells transfected with pEGFP alone (Figure 3B, lane 1). These results suggest that transiently increasing C/EBPα expression results in decreased endogenous PSA levels implying that C/EBPα may directly inhibit the transcription of the PSA gene and that the decrease in PSA may not be related to the reduced expression of AR.

### Further examination of the effects of C/EBPα expression on the transcriptional activity of PSA

**Figure 4:**
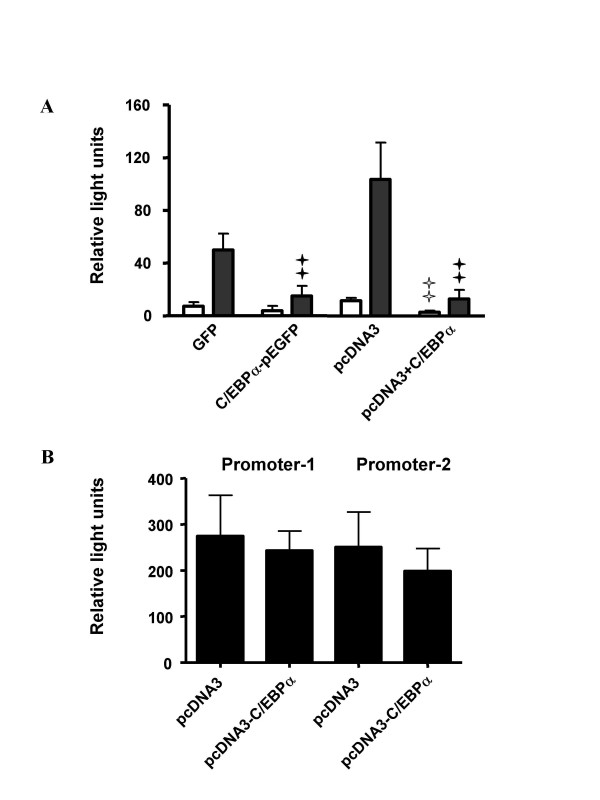
Inhibition of the activity of PSA promoter by C/EBPα. **A**. LNCaP cells were transiently co-transfected with a PSA promoter/enhancer-driven luciferase reporter plasmid and the C/EBPα expression plasmids, C/EBPα-pEGFP and pcDNA3-C/EBPα. After transfection, cells were treated for 36 hrs with 50 nM 5α-dihydrotestosterone (DHT) in RPMI 1640 medium supplemented with charcoal-stripped FCS. Cell extracts were collected with passive lysis solution, luciferase activity measured using pCMV renilla to standardize the transfection efficiency, and the results expressed as the mean relative light units ± standard deviation of 4 separate experiments. Open bars, basal transcription without DHT. Dark bars, transcription with DHT. Open double stars, statistically significant basal transcription in the C/EBPα expressing cells compared with control cells p-value < 0.01. Dark double stars, statistically significant DHT-stimulated transcription in the C/EBPα expressing cells compared with control cells with p-value < 0.01. **B**. Expression of C/EBPα did not inhibit the activity of PSMA promoter. The PMSA promoter-1(2 kb) and promoter-2 (5 kb) in the pGL-3 luciferase reporter plasmid, kind gifts from Dr. Sidney R. Grimes, were co-transfected with the C/EBPα expression plasmid into ALVA 101 cells. The luciferase assay was conducted as described in Methods.

To determine if the decreased expression of PSA was a direct effect of C/EBPα on PSA transcription we examined the effect of C/EBPα on expression of the PSA promoter. For these assays we used the PSA promoter coupled to a luciferase reporter gene. The PSA promoter consists of a proximal promoter containing two AREs and a distal enhancer with six AREs located about 4.2 kb upstream from the transcription start site. For these experiments, we used 5α-dihydrotestosterone (DHT), a nature ligand of androgen receptor, to activate AR signaling. LNCaP cells transfected with the PSA promoter/enhancer coupled to the luciferase reporter were co-transfected with C/EBPα-pEGFP or pcDNA3-C/EBPα in the presence and absence of DHT (Figure 4A). Both of the C/EBPα constructs significantly (p < 0.01) inhibited the PSA promoter although the pcDNA3-C/EBPα construct inhibited PSA transcriptional activity more than the C/EBPα-pEGFP construct both under basal conditions and in the presence of DHT. The greater inhibition by pcDNA3-C/EBPα could be the result of the C-terminus of the C/EBPα synthesized from the pcDNA3-C/EBPα construct being unencumbered by the EGFP moiety. The inhibition of PSA transcriptional activity in the presence of DHT also demonstrates that C/EBPα may affect AR signaling in activation of the PSA promoter/enhancer. To exclude the inhibition of the PSA promoter by C/EBPα as a non-specific artifact, we examined the promoter activity of prostate specific membrane antigen (PSMA) by co-transfection of the PMSA promoter with the C/EBPα expression plasmid. In contrast to the PSA promoter, the PSMA promoter was not inhibited by the expression of C/EBPα (Figure 4B). In addition, we examined the effect of the forced expression of C/EBPα in the PC3 and LNCaP retrovirus transduced cell lines on levels of granulocyte colony- stimulating factor (G-CSF) receptor mRNA. C/EBPα is well known to stimulate transcription of the G-CSF receptor and in the transduced PC3 and LNCaP cell lines we observed that forced expression of C/EBPα increased the RNA level of the G-CSF receptor by 2–6 fold (data not shown). Taken together these results suggest that inhibition by C/EBPα of the PSA promoter/enhancer activity is specific.

**Figure 5:**
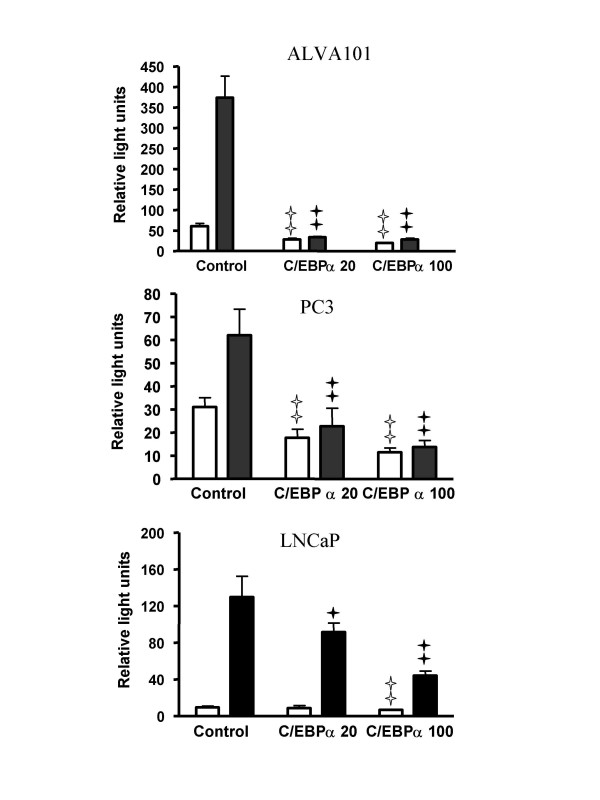
Dose-dependent inhibition by C/EBPα of the activity of PSA promoter/enhancer. Prostate cancer cells, ALVA101, PC3, and LNCaP cells were transiently transfected either with 0, 20, or 100 ng C/EBPα in the presence (dark bars) or absence (open bars) of DHT. Fifty ng of the androgen receptor expression vector (also the kind gift of Dr. Stephen P. Balk) was co-transfected into the AR negative cell lines, ALVA101 and PC3. The results are expressed as in Figure 4A with the double star representing a p-value < 0.01, a single star, p-value < 0.05, and the open and dark stars representing the same comparisons as in Figure 4A.

To exclude that the suppression of PSA transcription was specific to LNCaP cells we also examined the effect of pcDNA3-C/EBPα on the PSA promoter/enhancer in two other prostate cancer cell lines, ALVA101 and PC3, neither of which express AR or PSA. The ALVA101 and PC3 cells were co-transfected with an AR expressing plasmid to allow comparison of the effect of C/EBPα expression in the presence of DHT. As seen in Figure 5, in both the ALVA 101 and PC3 cells pcDNA3-C/EBPα significantly inhibited PSA transcription both in the absence and presence of DHT. Indeed, the inhibition of reporter expression was greater in the ALVA101 and PC3 cells than the LNCaP cells even with transfection with higher amounts of C/EBPα. In addition, the effect of C/EBPα on the proximal PSA promoter was also examined and demonstrated that in all three cell types, LNCaP, ALVA101, and PC3, that transient transfection with C/EBPα decreased proximal promoter transcription in the presence and absence of DHT although to a lesser degree than with the full-length promoter/enhancer (data not shown).

**Figure 6:**
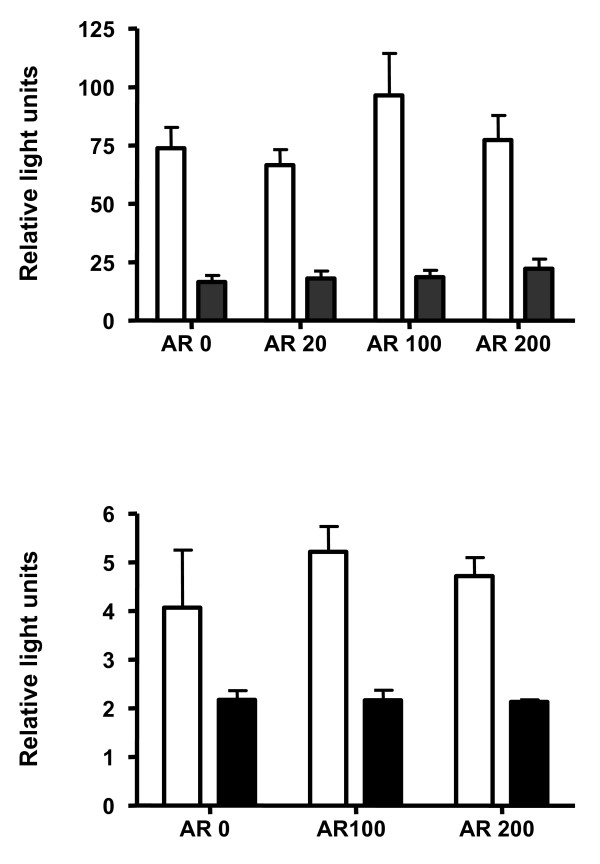
Increased androgen receptor expression does not block the inhibitory effect of C/EBPα on PSA promoter activity. LNCaP cells were co-transfected with 0, 10, 100, and 200 ng of androgen receptor cDNA in the presence (dark bars) or absence (open bars) of 100 ng cDNA for C/EBPα and in the presence of luciferase reporter construct driven by either the promoter/enhancer (upper panel) or PSA proximal promoter (lower panel). DHT was added at 50 nM and after 36 hrs the luciferase activity was measured. The results expressed as relative light units are the means ± standard deviation of 3 separate experiments.

To examine if C/EBPα acted directly on the AR and interfered with the function of the AR in androgen-dependent signaling in the enhanced inhibition by C/EBPα of the PSA promoter/enhancer in DHT-stimulated transcription, varying amounts of AR expression plasmid were co-transfected with luciferase reporters and C/EBPα expression vector into LNCaP cells and transcription activation was measured by luciferase activity (Figure 6). Alteration of the amount of AR expressed did not affect the inhibition by C/EBPα either of the full-length PSA promoter/enhancer (Figure 6, upper panel) or the proximal PSA promoter (Figure 6, lower panel). These results indicate that inhibition by C/EBPα of androgen-dependent transcriptional activation of PSA was independent of the levels of AR expression. In additional experiments, C/EBPα could not be co-immunoprecipitated with AR suggesting no direct interaction between AR and C/EBPα (data not shown).

**Figure 7:**
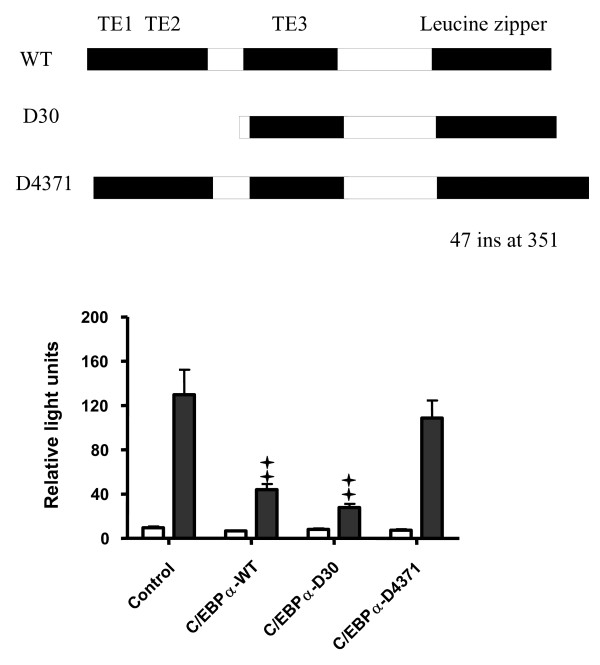
Effect of isoforms and a C-terminal mutation of C/EBPα on PSA promoter activity. LNCaP cells were transfected with PSA promoter/enhancer-driven luciferase reporter construct and expression vectors of C/EBPα expressing the wild type (WT) holoprotein, a mutated C/EBPα (D30) that gives rise to a protein identical to the p30 isoform of C/EBPα formed by an alternative initiation codon, and a C-terminal mutant (D4371) of C/EBPα formed by a 47 amino acid insertion at amino acid position 351. Luciferase activity was measured as in Figure 4. **A**. Schematic representations of the p42 and p30 isoform of C/EBPα and the C-terminal mutant protein (2). **B**. Expression of luciferase activity from the reporter construct after transfection with 100 ng of cDNA for C/EBPα p42 (wild type, WT), p30 isoform (D30), or C-terminal mutated cDNA (D4371) in LNCaP cells in the presence (dark bars) or absence (open bars) of DHT. The results expressed as relative light units are the means ± standard deviation of 3 separate experiments. Dark double stars signify statistical significance at p-value < 0.01 compared with control in DHT-stimulated transcription activity.

Mutations in the C/EBPα gene have been found in the myeloblasts of patients with acute myelogenous leukemia, FAB classification M2. Mutations have been observed to occur in both the transactivating and leucine zipper domains of the N- and C- termini portions of the gene, respectively. The protein products of the mutated C/EBPα act as dominant negative suppressors of wild type C/EBPα and block the stimulation of myeloid target genes [2]. We examined the effect of representative mutations of C/EBPα on PSA-luciferase activity (Figure 7). The mutation designated D30 showed considerable inhibition of PSA-luciferase activity while D4371, a mutation occurring in the leucine zipper domain exhibited minimal affect on C/EBPα inhibition of the reporter construct. These results suggest that the C-terminal leucine zipper region of C/EBPα is more important than the transactivating domain in the suppression of the PSA promoter/enhancer.

**Figure 8:**
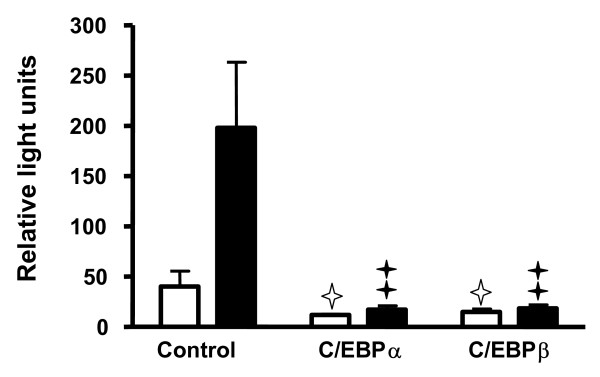
Inhibition of promoter/enhancer activity of PSA by human C/EBPα and C/EBPβ. The LNCaP cells were transfected either with human C/EBPα or C/EBPβ and the full-length PSA promoter coupled to the luciferase reporter. Luciferase activity was measured in the presence (dark bars) or absence (open bars) of DHT as described in Figure 4. The results expressed as relative light units are the means ± standard deviation of 3 separate experiments. The open and dark double stars represent P values as described in Figure 4A.

Analysis of the amino acid sequence of the members of C/EBP subfamily shows that the C-terminal leucine zipper region is highly conserved. Hence, if the C-terminus is essential for inhibition of the PSA promoter/enhancer, than other members of the C/EBP subfamily should exhibit a similar effect. We tested this hypothesis by transfecting LNCaP cells with C/EBPβ and demonstrated that human C/EBPβ, as with human C/EBPα, significantly inhibited PSA promoter/enhancer activity (Figure 8). The inhibition by human C/EBPβ was 91% in DHT-stimulated transcription and 63% in basal transcription, respectively, compared to C/EBPα which caused inhibition of 94% and 57%, respectively.

## Discussion

C/EBPα is expressed in many tissues including white and brown adipose tissue, myeloid cells, lung, prostate, ovary, and colon [11,30]. The role of C/EBPα in adipose, myeloid, and hepatocyte terminal differentiation has been well established. In myeloid cells, for example, the expression of C/EBPα is regulated and C/EBPα expression leads, in turn, to expression of genes necessary for cell differentiation and to limited myeloid proliferation [4,7,31,32]. However, all hematopoiesis is not affected similarly. While C/EBPα stimulates the differentiation of myeloid cells, such as granulocytes, increased expression of C/EBPα significantly blocks the differentiation of erythroid precursors [6]. The regulation by C/EBPα in proliferation and differentiation might contribute to the development of the malignant phenotype. The aberrant expression of C/EBPα has been described in myeloid leukemias [10,13]. In a subset of myeloid leukemias mutations of C/EBPα result in the synthesis of abnormal proteins that act as dominant negative proteins to block the action of C/EBPα with a resulting block of differentiation and loss of the limits on proliferation [2]. In other leukemias the fusion protein formed from the AML1-ETO translocation down-regulates transcription of the C/EBPα gene [10].

However, only to varying extents has the role of C/EBPα in the differentiation of some of the other tissues been defined. To even a lesser extent has the presence of aberrant expression of C/EBPα in malignancies of these various organs been described. For example, C/EBPα has a gradient of expression in the small intestine of mice with the greatest expression in the most proximal portions of the intestine and without any expression detected in mouse colonic epithelium [33]. In the mouse intestine C/EBPα was found in the epithelium of the villi and not in the crypt cells suggesting that here too C/EBPα expression is associated with terminal differentiation. In human colon and colonic cancers, however, C/EBPα can be detected suggesting that there is some species specificity and a slight decrease of expression has been detected in more advanced colon cancer [34]. In the rat ovary expression of C/EBPα increases with differentiation of the follicular cells [35]; to date there are no reports of altered C/EBPα expression in ovarian cancer. In addition to the detection of mutations in C/EBPα in acute myelogenous leukemia of subtype FAB classification M2, mutations have been detected in myelodysplastic disorders but only silent mutations have been detected in non-hematologic tumors including one lung cancer and one prostate cancer [13]. In the lung C/EBPα is expressed in the basal layer of normal lung tissue and expression of C/EBPα is recognized to contribute to type II cell differentiation [36]. However the role of expression of C/EBPα on the differentiation of lung cells is still unknown.

The studies presented here provide additional information to the study that examined C/EBPα expression by gene array [14] and in which expression of C/EBPα RNA was increased by three fold in cancerous prostate epithelium versus normal epithelium. C/EBPα expression at the protein level in malignant and non-malignant prostate glands has not been previously investigated. In the normal prostate, C/EBPα is predominately expressed in the basal layers of the epithelium with little C/EBPα detected by immunohistochemistry in the more differentiated secretory epithelium expressing AR and PSA. This is a distinctly different distribution than seen in other tissues where C/EBPα is expressed in differentiated cells. It is interesting that in our immunohistochemistry studies, p63 was found to co-localize with the expression of C/EBPα in normal prostate gland. p63 is a marker of the prostate gland basal layer cell and a potential marker for prostate stem cells, which are characterized by active cellular proliferation [37,38]. Further, in prostate cancer the detection of C/EBPα in the basal layer was lost, but C/EBPα staining could be detected in cancerous cells throughout pseudoglandular structures. In advanced prostate cancer with the loss of glandular like structures, C/EBPα was still expressed in some cancer cells with a similar intensity of staining as in the non-malignant epithelium. Interestingly, in the immunohistochemical staining of prostate tissue arrays for C/EBPα and AR, the ratio of C/EBPα to androgen receptor expression increased with increasing Gleason scores (data not shown) supporting our observation that forced overexpression of C/EBPα in LNCaP cells induced loss of AR expression.

To address the questions derived from our immunohistochemical staining, we first selected the PSA promoter/enhancer as a molecular marker to investigate the role of C/EBPα in the prostate. PSA seemed a reasonable choice: PSA is as a marker of prostate differentiation and is expressed in the terminally differentiated epithelium lining the lumen of tubules in the normal prostate gland as is AR; expression of PSA is androgen-dependent; and increased levels of PSA in the serum is an important biomarker for prostate cancer. The observation that C/EBPα was expressed in the basal epithelium suggested that C/EBPα may suppress PSA expression. In view of very low level of constitutional expression of C/EBPα protein in the LNCaP cells, a widely used prostate cancer cell line that expresses AR and PSA, exogenous C/EBPα was expressed by use of a retrovirus system and two pooled clones of LNCaP cells with stable expression of C/EBPα were established. Clone1 was found to express neither AR nor PSA immediately upon establishment of the clone. Clone 2 developed epigenetic loss of AR and PSA expression with serial passage. Given the rapidity in which AR expression is lost it is unlikely that the retrovirus itself caused the loss of AR expression. In addition, we have examined AR and PSA expression in LNCaP cells stably transfected with pcDNA3-C/EBPα and also noted decreased expression of PSA and AR (data not shown). However, the stable expression of C/EBPα introduced with a non-virus vector could not be maintained in LNCaP cells despite continued selection pressure with geneticin.

In the LNCaP clones with stable expression of C/EBPα, concomitant with the expression of C/EBPα we observed the loss of AR expression. Both C/EBPα and AR are important for the control of growth of prostate cells. The continued growth of the clones overexpressing C/EBPα itself was a surprise. In lung cancer cells and other cell types restoration or increased expression of C/EBPα caused growth arrest [36]. In contrast, in the LNCaP clones expressing C/EBPα we did not observe any signs of growth arrest. The resistance to C/EBPα-induced cell growth arrest may have taken place during clonal selection. However, the establishment of stable expression of C/EBPα itself in several prostate cell lines meant that cells could escape from the negative regulation of cell proliferation by C/EBPα. This may explain the difference of cell fates between transient and stably expression of C/EBPα. A recent similar example has been observed in hepatocytes where the suppression of cell proliferation by C/EBPα is lost during oncogenesis by dephosphorylation of C/EBPα which leads to failure of C/EBPα to interact with cdk2 and E2F as well as sequestering retinoblastoma protein [39,40]. Similar loss of the interaction between C/EBPα and cell cycle related proteins was found in established prostate cell lines (date not shown). In addition, the expression of C/EBPα in the actively proliferating basal cell layer of the prostate suggests that C/EBPα does not cause growth arrest in normal prostate epithelium.

The loss AR expression occurs in several prostate cancer cell lines, such DU145, PPC1 and PC3, and occurs frequently during the clinical evolution of prostate cancer [41,42]. The data from gene expression profiles shows that expression of AR RNA is absent in 30 to 40 % of prostate cancer samples (GEO accession: GSE1431) and the proportion of prostate cancers not expressing AR is increased in metastatic prostate cancer (GEO accession:GSE3325). LNCaP cell lines with loss of AR expression exhibit stronger tumorgenicity and a greater metastatic potential in xenografts than AR positive LNCaP cells [43]. In another prostate cancer cell line that lacked AR expression restoration of that expression restored AR-dependent cell growth [42]. We would predict that the loss of AR in our C/EBPα overexpressing LNCaP clones would cause a transformation of cells from androgen-dependent to androgen-independent growth and that the cells would exhibit more aggressive growth, invasion, and metatstatic potential. Indeed, our initial analysis of cell growth showed a more rapid growth of the C/EBPα expressing Clone 2 LNCaP cells compared with the control cells at passage 8 when AR was barely detectable. In addition, our immunohistochemical findings of reduced expression of AR in advanced prostate cancer specimens especially those that express C/EBPα is in agreement with recent demonstrations of altered AR expression especially in androgen-independent prostate cancer [44].

The mechanism(s) for the loss of AR expression both in prostate cell lines and prostate cancer tissue are not completely understood and expression may vary in the same patient at different sites of recurrence [44]. Methylation of the AR gene promoter has been reported in advanced hormone-independent prostate cancer tissue [41] and the addition of 5-Aza-2'-deoxycytidine(5-AZA) into DU145 cells with a heavily methylated AR promoter restored AR mRNA expression [45]. In our C/EBPα overexpressing LNCaP cells, treatment with 5-AZA at 10 μM increased expression of AR and PSA in early passages of clone 2 but not clone 1 cells (data not shown). However, increased expression of AR by 5-AZA was seen in the corresponding control cells suggesting that methylation affecting AR expression was occurring in both C/EBPα expressing and non-expressing cells.

The immunohistochemical result demonstrating C/EBPα expression in the basal layer of the prostate epithelium with no expression of PSA in these cells led us to examine the effect of C/EBPα on the expression of the PSA promoter/enhancer. The inhibition of expression was not cell type specific as similar studies in CV1 and COS7 cells of co-transfection of C/EBPα and PSA promoter reporter gene demonstrated similar results (data not shown). A recent report shows that inhibition of PSA expression in LNCaP cells was dependent on an interaction between AR and C/EBPα [46] while our results indicated that the inhibition was independent of any direct interaction with AR. With transient expression of C/EBPα in prostate cells, ALVA 101 and the non-prostate cell line CV-1 we could not show inhibition of the MMTV promoter which contains a cryptic AR (data not shown). Nor did we observe that an increased expression of AR affected the suppression of the PSA promoter by C/EBPα. In addition, a standard search for transcription factor binding sites by TESS or TFSEARCHING showed that more than 10 potential C/EBPα binding sites in the PSA promoter. Preliminary studies with a gel shift assay indicated that C/EBPα could bind to at least 3 of these sites. Therefore, direct binding of C/EBPα to PAS promoter may explain the suppression of the PSA promoter by C/EBPα. We can not exclude that C/EBPα might also stimulate expression of coactivators. On the other hand, the results also suggest that the C-terminal leucine zipper region was more important than the transactivating domains.

We are beginning to explore, amongst the various genes whose expression in prostate cancer cells is uniformly altered by forced expression of C/EBPα, those genes that may contribute to the loss of AR expression and which may provide therapeutic targets for the difficult clinical problem of hormonal independence.

## Conclusion

C/EBPα was differentially displayed between normal and cancerous prostate. Long term of stably expression of C/EBPα in prostate cancer cells stimulates cell proliferation. Down-regualtion of PSA expression by C/EBPα depends on the loss of AR expression in AR posive LNCaP cells and on the universal inhibiton of PSA promoter.

## Competing interests

The author(s) declare that they have no competing interest.

## Authors' contributions

HY participated in development of concept and design, performed experiments, analyzed data, draft manuscript. HSR and DGT were involved in critically revising the manuscript. JG contributed to idea development, data interpretation, manuscript revision, and final approval. All authors read and approved the manuscript.

## Pre-publication history

The pre-publication history for this paper can be accessed here:
